# Computer-analyzed facial expression as a surrogate marker for autism spectrum social core symptoms

**DOI:** 10.1371/journal.pone.0190442

**Published:** 2018-01-02

**Authors:** Keiho Owada, Masaki Kojima, Walid Yassin, Miho Kuroda, Yuki Kawakubo, Hitoshi Kuwabara, Yukiko Kano, Hidenori Yamasue

**Affiliations:** 1 Department of Child Psychiatry, School of Medicine, The University of Tokyo, Tokyo, Japan; 2 Department of Psychiatry, Hamamatsu University School of Medicine, Hamamatsu, Japan; Chiba Daigaku, JAPAN

## Abstract

To develop novel interventions for autism spectrum disorder (ASD) core symptoms, valid, reliable, and sensitive longitudinal outcome measures are required for detecting symptom change over time. Here, we tested whether a computerized analysis of quantitative facial expression measures could act as a marker for core ASD social symptoms. Facial expression intensity values during a semi-structured socially interactive situation extracted from the Autism Diagnostic Observation Schedule (ADOS) were quantified by dedicated software in 18 high-functioning adult males with ASD. Controls were 17 age-, gender-, parental socioeconomic background-, and intellectual level-matched typically developing (TD) individuals. Statistical analyses determined whether values representing the strength and variability of each facial expression element differed significantly between the ASD and TD groups and whether they correlated with ADOS reciprocal social interaction scores. Compared with the TD controls, facial expressions in the ASD group appeared more “Neutral” (*d* = 1.02, *P* = 0.005, *P*_FDR_ < 0.05) with less variation in Neutral expression (*d* = 1.08, *P* = 0.003, *P*_FDR_ < 0.05). Their expressions were also less “Happy” (*d* = −0.78, *P* = 0.038, *P*_FDR_ > 0.05) with lower variability in Happy expression (*d* = 1.10, *P* = 0.003, *P*_FDR_ < 0.05). Moreover, the stronger Neutral facial expressions in the ASD participants were positively correlated with poorer ADOS reciprocal social interaction scores (*ρ* = 0.48, *P* = 0.042). These findings indicate that our method for quantitatively measuring reduced facial expressivity during social interactions can be a promising marker for core ASD social symptoms.

## Introduction

Deficits in social communication and interaction are core symptoms of autism spectrum disorder (ASD), and includes abnormalities in nonverbal communicative behaviors such as eye contact, gestures, voice prosody, and facial expressions [1]. Treatments for ASD include pharmaceuticals for reducing behavioral problems such as irritability [2, 3] and a few novel compounds under development are expected to ameliorate ASD core symptoms [2, 4] on the basis of their effects on a number of behavioral and neural markers for social deficits in ASD at experimental settings [5, 6]. However, clinical evidence that novel therapeutics improve ASD core symptoms is limited, at least partially because valid, reliable, and sensitive longitudinal outcome measures that detect clinical changes in ASD core symptoms over time have yet to be established [6, 7].

The Autism Diagnostic Observation Schedule (ADOS) [8] and Autism Diagnostic Interview-Revised (ADI-R) [9] are the gold standards for assessing ASD core symptoms. Several studies have successfully used the ADOS to measure intervention effectiveness in ASD [10–12], and this is likely because its semi-structured design and specificity allow reproducible evaluation of ASD core symptoms by eliciting them implicitly during social interactions. However, the ADOS and other currently available diagnostic tools were not originally formulated to assess longitudinal changes. For example, repeating certain questions at short intervals would be inappropriate, and administering the complete exam takes considerable time and can impose a certain degree of psychophysical burden on participants.

Characterizing the facial expressions of people with mental disorders, in particular with schizophrenia or depression [13], has been of clinical interest in recent years, and has been investigated using standardized measures including electromyography (EMG) and specific coding systems such as the Facial Action Coding System (FACS) [13, 14]. However, although a large number of studies have reported difficulties in recognizing emotional faces in individuals with ASD [15], few have focused on quantitative measurement of the deficits these individuals have in expressing emotions with their own faces [13]. Most case-control studies of facial expression in ASD have used various means to elicit emotional facial expressions during observation [16–24], and have assessed them with manual ratings based on established coding systems (e.g., FACS) [20, 21] or other specially developed subjective rating scales [16, 23, 24], time measurements [22, 25], or facial EMG [17–19]. While these studies imply that individuals with ASD show a reduction in certain elements of facial expressions under specific conditions, to our knowledge, no study has comprehensively evaluated spontaneous facial expressions with objective measurements, especially during implicitly evoked natural social interaction.

The current study tested the hypothesis that atypical features in facial expression during social interaction could be used to grade autistic deficits in social reciprocity. We employed computerized quantitative facial expression analysis (FEA) [26, 27] as an alternative approach to conventional measures to obtain an objective, quantitative measurement for facial expression. This allowed emotional elements to be evaluated all at the same time instead of categorizing them into one representative emotional face. Because of its established reliability and validity, the FEA was based on observational situations taken from activities in the ADOS. Therefore, the current study included the following steps: (1) apply computerized quantitative FEA on appropriate parts of the ADOS to characterize autistic features of facial expression in participants with ASD; (2) compare these characteristics with those for typically developing (TD) controls; (3) test associations between the identified autistic features in facial expression and ADOS reciprocal social interaction scores; (4) evaluate whether the identified facial expression measurements are collaterally related to other clinical or demographic indices.

## Materials and methods

### Participants

Eighteen high-functioning Japanese adult males with ASD from the outpatient clinic of The University of Tokyo Hospital participated in the study. Diagnostic and clinical assessments have been described in detail elsewhere [12]. Briefly, an experienced psychiatrist (H.Y.) made the diagnoses according to the strict criteria of the Diagnostic and Statistical Manual-Revision IV-Text Revision [28] with more than two months of follow-up examinations. Other certified psychiatrists/psychologists confirmed the diagnosis using the ADI-R [9] (H.K.) and ADOS Module 4 [8] (M.K.). The inclusion criteria for participants with ASD were: (1) a diagnosis of ASD, (2) male gender, (3) full-scale IQ > 80 on the Wechsler Adult Intelligence Scale-Revised, Japanese version (WAIS-R) [29], and (4) aged 18–55 years. Exclusion criteria were: history of seizures, traumatic brain injury with any known cognitive consequences, loss of consciousness for more than 5 minutes, substance abuse/addiction, and unstable comorbid psychiatric symptoms.

Seventeen Japanese adult males with TD were recruited as control participants. All were screened by two separate psychiatrists (H.Y. and K.O.) for the presence or past history of neuropsychiatric disorders using the Structured Clinical Interview for DSM-IV Axis I Disorder [30], and for a history of neuropsychiatric disorders in their first-degree relatives.

The ethical committee of University of Tokyo Hospital approved this study (10245). After a complete explanation of the study, every participant’s mental capacity to consent was confirmed by a psychiatrist (H.Y. or K.O.), and written informed consent was obtained from all participants.

### Clinical assessments

In addition to the assessments for eligibility such as WAIS-R, ADI-R, and ADOS, the following indices were obtained for all participants: self-/parental-socioeconomic status (SES) [31], World Health Organization Quality-of-Life questionnaire (WHOQOL) [32], Global Assessment of Functioning (GAF) [33], Autism Spectrum Quotient (AQ) [34], State and Trait Anxiety Inventory state (STAI-state) [35], and Center for Epidemiologic Studies Depression Scale (CESD) [36].

The verbal IQs for the TD participants were estimated using the Japanese version [37] of the National Adult Reading Test (NART) [38]. While the NART can estimate the verbal IQs of TD individuals, this estimation can be problematic for those with ASD because of their imbalanced intellectual abilities, as well-known discrepancies between subscales of the WAIS-R. Therefore, the IQs of the participants with ASD were assessed using the WAIS-R.

### ADOS administration

The ADOS Module 4 for verbally fluent adults [8] was administered to the ASD group in a standard order by one of three administrators (H.Y., H.K., or Y.K.) who had completed a training course for research use of the ADOS, and who had been validated by another certified administrator (M.K.). Each administration was recorded on video, and the certified administrator (M.K.) rated the ADOS scores from the videos to minimize inter-rater variability. The ADOS has 15 activities (*Construction task*, *Telling a story from a book*, *Description of a picture*, *Conversation*, *Current work/school*, *Social difficulties/annoyance*, *Emotions*, *Demonstration task*, *Cartoons*, *Break*, *Daily living*, *Friends/marriage*, *Loneliness*, *Plans and dreams*, and *Creating a story*), one of which (see **Situation selection out of the ADOS activities**, below) were administered to TD individuals (by H.Y.) in the same manner and settings as were used for the individuals with ASD. Footage of TD individuals was also recorded.

### Video recording and editing

A video camera (Handycam HDR-CX180, Sony Corporation, Tokyo, Japan, 2011) was mounted on a one-meter tripod approximately two meters in front of the participant. After the participant was seated, the administrator adjusted the camera angle and moved to sit at a right angle to the participant so that the video frame included the full face of the participant and the side of the administering author’s face throughout procedure. Only when the participant stood up to narrate alone during the *Cartoons* activity did the administrator stand behind the camera and temporarily adjust the camera angle to keep the full face of the participant in the center of the frame. The camera angle was returned back to its original position immediately after this activity.

The video files were 1920 × 1080/60i with H.264/MPEG-4 AVC compression. To optimize the sensitivity and accuracy of the FEA, we segmented the video clips such that each clip contained one ADOS activity, and then cropped them to a 960 × 540 resolution that focused on the face region. If necessary, we minimally corrected the brightness/contrast.

### Computerized facial analysis and data processing

We used commercially available software dedicated to FEA for quantifying facial expressions (FaceReader version 6.1; Noldus Information Technology Inc., Wageningen, The Netherlands). This software outputs time-series datasets comprising expression intensity (EI) values between zero and one for each of seven facial expression elements (Neutral, Surprised, Happy, Sad, Scared, Angry, and Disgusted) [14]. At the same time, it estimates head orientation with reference to the front-facing head position and outputs its three elemental Euler angles, which are defined by rotations about the axes of a spatial coordinate system.

The methods and algorithms the software uses for FEA are detailed in the FaceReader version 6.1 Reference Manual (Noldus Information Technology Inc., Wageningen, The Netherlands, 2015). The software provides five face models (General, General61, Children, East Asian, and Elderly) that correspond to the datasets used in algorithm training. We employed the “East Asian” face model not only according to the software specifications, but also based on a preliminary experiment with a small convenient sample. By comparing participant EI-value profiles for each model with our impression of their facial emotions, we concluded that the “East Asian” face model was indeed the most suitable for adult Japanese males. Additionally, we used the “participant calibration” function provided by the software to minimize biases in facial expression that result from a participant’s own natural countenance and the camera/lightning conditions. According to the Reference Manual, this method removes biases in emotional expression intensities but does not increase the intensities. As per calibration instructions, the first part of each video clip was used as neutral picture for calibration.

Although the software automatically detects faces and evaluates their expressions using internal algorithms[26], the whole data-acquisition process was monitored by a single operator (K.O.). Settings for face-detection sensitivity and accuracy were minimally adjusted to maintain the quality of face recognition during this process. When the time-series output was fragmentary due to frequent failures in face recognition, problematic video sequences shorter than one second was ignored to maintain data reliability. The degree of successful face recognition was evaluated for every video clip, and those with a low proportion of successful frames (< 20%) were excluded from further analyses [27].

### Situation selection out of the ADOS activities

To ensure a uniform and consistent setting for observing facial expressions, a subset of the 15 ADOS activities were selected based on their degree of structure (to orient a participant to a certain consistent response), repeatability (to elicit improvised responses), and feasibility (whether faces were remained positioned such that the software could correctly recognize facial expressions; whether the activity length was short enough to observe a situation-evoked facial expression with minimal contamination).

First, the structure and repeatability of each ADOS activity was qualitatively evaluated (S1 Table), and the *Construction task*, *Telling a story from a book*, *Demonstration task*, and *Cartoons* activities were selected. Among these activities, *Demonstration task* was excluded because this activity requests the participant to demonstrate brushing his teeth, which might contaminate the facial expression.

Second, we quantitatively evaluated the feasibility of these three candidate activities using summed Z-scores for the following four indices: activity length, proportion of successfully recognized faces, and validity (i.e., bias of distribution) and reliability (i.e., consistency of distribution) of the head orientations, with reference to the front-facing position. The head orientation of each participant in an activity was represented by the mode vector of the time-series Euler angles (i.e., the most probable head orientation during the activity), which was calculated by multivariate kernel density estimation using Gaussian kernels. The distribution of head orientations was described by their corresponding vectors and put into a three-dimensional coordinate system; thereafter, their validity was evaluated by the length of vectors (equal to Euclidean distances from the origin), and their reliability was evaluated by Euclidean distances between the center of gravity for the vectors in each activity and the end points of the vectors. Z-scores for these four values were plus-signed in favorable directions (i.e., shorter activity length, higher proportion of success in face recognition, shorter vector length, and shorter distance between the center of gravity and the end point of vector), and summed for each participant in each activity.

Then, we identified the ‘best’ activity as the one with the highest mean sum of Z-scores, and excluded any activities with mean sums of Z-scores that were significantly lower than that for the ‘best’ activity (one-tailed *T*-tests, FDR corrected *P* < 0.05). We focused analysis on the ‘best’ activity, and then the remaining activities, if any.

### Facial expression data-processing and statistical analyses

Data processing and statistical analyses were conducted using IBM SPSS 22.0 (IBM Corp., Armonk, NY), MATLAB R2015a (The MathWorks, Natick, MA), R 3.3.2 (http://www.R-project.org/), and Python libraries for scientific computation (NumPy, and SciPy) [39].

Time-trend datasets from the selected activities of each participant yielded two pairs of EI variables, which represent the distribution of EI values for each facial expression element: the first comprised the time-averaged mean (Mean) and standard deviation (SD), and the second comprised the value at which the probability value was maximum (Mode) and the natural logarithm of the maximum probability value (LogP) on the probability density function that was estimated by kernel density using Gaussian kernels. Considering the close relationship between these two pairs, correlations between Mode and Mean (both represent the “strength” of the facial expression element), and between LogP and SD (both reflect the “variability” of the element) were evaluated for each facial expression element using Spearman’s rank correlation (significance level was set at FDR-corrected *P* < 0.05). If we detected robust correlations, we planned to use the Mode/LogP pair as the main outcome measurements, as opposed to the Mean/SD pair. This is because the Mode is less likely to be influenced by outliers, it is considered more stable than the Mean for representing a “typical” EI value during observation of facial expressions. (S1 Data set)

### Comparison between the ASD and TD groups

To determine whether EI values for the seven facial expression elements differed between groups, we employed a repeated-measures analyses of variance (ANOVA) with EI value as the dependent variable, ADOS activity as the within-participants factor, and group (ASD / TD) as the between-participants factor. A post-hoc analysis was planned to evaluate significant interactions with two-tailed *T*-tests. If only one ADOS activity was selected, groups were compared with two-tailed *T*-tests on each EI variable. Statistical significance was set at false discovery rate (FDR)-adjusted *P* < 0.05 [40].

### Correlation analyses between the EI variables identified with autistic features and clinical/demographic indices

As the EI variables that were determined to differ between ASD and TD groups could be expected to reflect autistic deficits in social reciprocity, we analyzed their correlations with ADOS reciprocal social interaction scores using Spearman’s rank correlation with a statistical significance of *P* < 0.05.

Additionally, to identify other clinical correlates of the identified autistic features in facial expression, we evaluated the correlations between these EI variables and the following clinical indices: CESD, STAI-state, WHOQOL, GAF, and AQ for both groups; ADI-R qualitative abnormalities in reciprocal social interaction, qualitative abnormalities in communication, restricted, repetitive, and stereotyped patterns of behavior scores; and ADOS communication, stereotyped behaviors and restricted interests scores for the ASD group. Further, to detect potential confounding factors that could affect our conclusions regarding autistic features in facial expression, we analyzed the correlations between the EI variables and the following demographics: age, height, weight, self-/parental-SESs, and verbal/performance/full IQs. These correlations were evaluated separately for each group with consideration for differences in data distribution. Spearman’s rank correlation was employed and the significance level was set at *P* < 0.05 with FDR correction [40].

## Results

### Demographics

All participants with ASD exhibited normal-to-high intelligence in the full-scale IQ. One participant with ASD was on medication with serotonin and norepinephrine reuptake inhibitor for depression but in remission of it, while the others were free from any medication. The ASD and TD group did not differ in age, height, body weight, parental SES, or verbal IQ. The ASD group showed significantly higher scores on the self-SES, AQ, and CESD, and significantly lower scores on the GAF and WHOQOL than the TD group (*P* < 0.05), indicating that people with ASD tended to have low social or educational level, high autistic/depressive traits, and poor well-being (Table 1).

**Table 1:** Demographic characteristics of participants with autism spectrum disorder (ASD) and those with typical development (TD).

	ASD (N = 18)		TD (N = 17)		
Variable	Mean	SD	Mean	SD	*P*-value
Age (range)	32.2 (24−43)	7.0	29.6 (23−34)	4.3	0.22
Height, cm	168.5	4.3	171.6	4.3	0.051
Body weight, kg	65.9	14.7	62.4	7.1	0.38
Socioeconomic status ^*1*^	2.7	0.9	1.2	0.4	< 0.001 ^*4*^
Parental socioeconomic status ^*1*^	2.2	0.6	1.9	0.5	0.087
Full-scale IQ ^*2*^	105.8	10.9			
Verbal IQ ^*2*^	112.5	12.1	117.7	6.1	0.14
Performance IQ ^*2*^	94.1	15.3			
CESD	19.2	11.3	9.1	10.6	0.012 ^*4*^
STAI state anxiety	47.4	14.9	38.5	10.0	0.051
WHOQOL	3.0	0.8	3.7	0.4	0.002 ^*4*^
GAF	46.6	5.3	81.5	5.3	< 0.001 ^*4*^
AQ	35.7	5.5	18.0	6.5	< 0.001 ^*4*^
ADI-R					
Qualitative abnormalities in reciprocal social interaction	14.8	6.1			
Qualitative abnormalities in Communication	11.6	4.1			
Restricted, repetitive, and stereotyped patterns of behavior	4.0	2.4			
ADOS					
Reciprocal social interaction	8.1	1.7			
Communication	3.7	1.4			
Stereotyped behaviors and restricted interests	0.4	0.5			
Video length, sec ^*3*^	64.6	26.0	49.2	11.8	0.038 ^*4*^
Proportion of success in face recognition in video frames, % ^*3*^	86.7	22.4	91.3	11.8	0.46

^*1*^Assessed using the Hollingshead two-Factor Index of Social Position [31], in which a higher score indicates a lower status.

^*2*^The IQs of participants with ASD were measured using the Wechsler Adult Intelligence Scale. The verbal IQ of those with TD was estimated using the Japanese version of National Adult Reading Test.

^*3*^Measured for the video clips recording the *Cartoons* activity, which was the only ADOS activity selected for facial expression analysis.

^*4*^Significant difference between both groups by two-tailed *T*-test (*P* < 0.05).

Abbreviations: *IQ*, intelligence quotient; *CESD*, Center for Epidemiologic Studies Depression Scale; *STAI*, State and Trait Anxiety Inventory; *WHOQOL*, World Health Organization Quality-of-Life questionnaire; *GAF*, Global Assessment of Functioning; *AQ*, Autism Spectrum Quotient; *ADI-R*, Autism Diagnostic Interview-Revised; *ADOS*, Autism Diagnostic Observation Schedule; *SD*, standard deviation

### Activity selection based on feasibility

We successfully obtained 54 video clips from the 18 individuals with ASD, and first used them to select the best ADOS activity such as *Construction task*, *Telling a story from a book*, or *Cartoons* for each participant. Three out of the 54 video clips were excluded from this feasibility evaluation because participants blocked their faces with their hands, causing the proportion of video frames with successful face recognition to be too low (< 20%; *Construction task* for one participant; *Telling a story from a book* for two).

Of the three ADOS activities, *Cartoons* yielded the highest mean sum of Z-scores (2.25 ± 1.99) for video length, proportion of successful face recognition, and validity and reliability of head orientation. The other two activities yielded significantly lower mean sums of Z-scores (*Construction task*: −0.69 ± 1.13, *t*_33_ = 5.25, *P* < 0.001, *P*_FDR_ < 0.05; *Telling a story from a book*: −1.79 ± 2.89, *t*_32_ = 4.55, *P* < 0.001, *P*_FDR_ < 0.05; S2 Table). Therefore, only the *Cartoons* activity was used for the quantitative FEA.

### Data description of facial expressions in the extracted ADOS activity

None of the footage of the *Cartoons* activity for any of the participants was excluded due to low face recognition (< 20%). Although the length of the activity was significantly longer in the ASD group than in the TD group (*P* = 0.038), the proportion of face-recognition success did not significantly differ between the groups (Table 1). Further, neither the length of activity nor the proportion of face-recognition success was significantly related to any demographic factor including age, height, body weight, or self-/parental-SES, IQ in either group (*P* > 0.05).

For each of the seven facial expression elements, the Modes of the EI values were significantly and positively correlated with their Means, and their LogP values were significantly and negatively correlated with their SDs (*P*_FDR_ < 0.05; S3 Table). Therefore, we chose Mode and LogP to be the main outcome measurements. We also performed supplemental statistical analyses using the Mean/SD pair and these results are shown in the Supporting Information (S4 Table and S2−S4 Figs).

### Comparison of EI variables between ASD and TD groups

**Fig 1:**
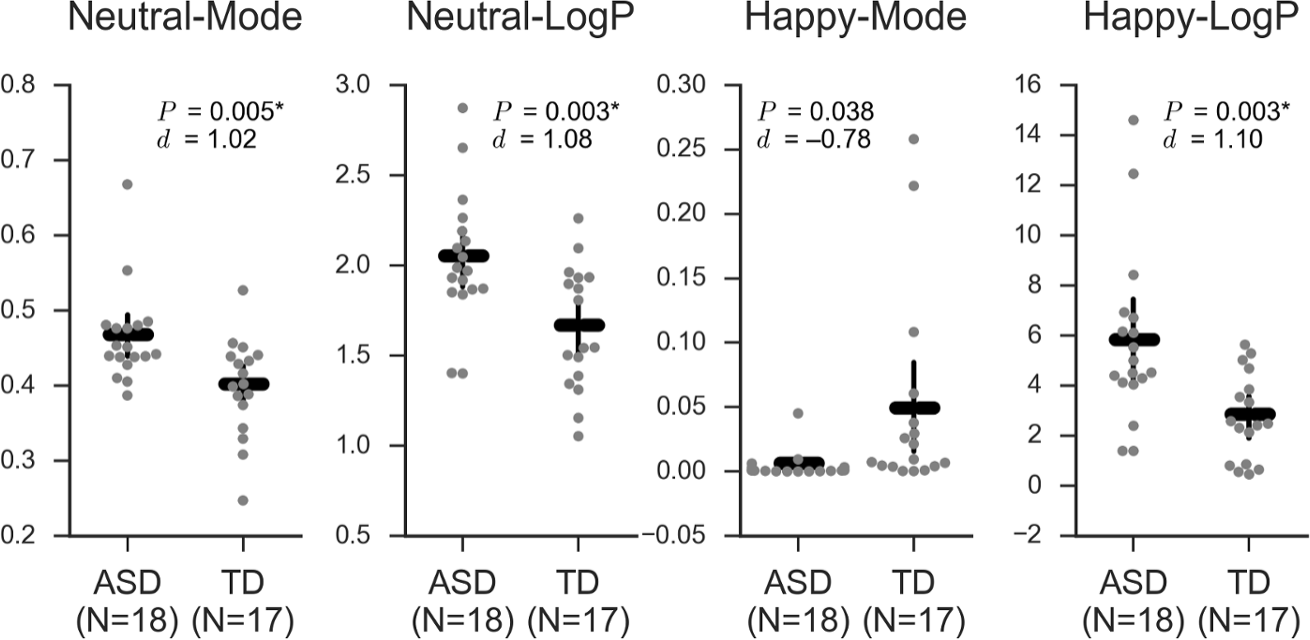
Comparison of EI variables between the autism spectrum disorder (ASD) and typical development (TD) groups. Only Mode/LogP of Neutral/Happy EI values are shown here. Neutral-Mode, Neutral-LogP, and Happy-LogP were significantly higher in the ASD group than in the TD group, and the effect sizes were large (*P*_FDR_ < 0.05, *d* > 1). The ASD group also showed a tendency for lower Happy-Mode (*P* < 0.05, *P*_FDR_ > 0.05). Each dot represents each participant. The horizontal and vertical bars show mean values and 95% confidence intervals, respectively. *d*, Cohen’s *d*. *, *P*_FDR_ < 0.05. Abbreviations: *EI*, expression intensity; *LogP*, natural logarithm of the probability at the mode of the probability density function.

Compared with the EI variables in the TD group, the ASD group showed significantly higher Neutral-Mode (*t*_33_ = 3.03, *d* = 1.02, *P* = 0.005, *P*_FDR_ < 0.05), Neutral-LogP (*t*_33_ = 3.21, *d* = 1.08, *P* = 0.003, *P*_FDR_ < 0.05), and Happy-LogP (*t*_33_ = 3.30, *d* = 1.10, *P* = 0.003, *P*_FDR_ < 0.05). Additionally, the TD group tended to have lower Happy-Mode values (*t*_33_ = −2.26, *d* = −0.78, *P* = 0.038, *P*_FDR_ > 0.05) (Table 2 and Fig 1). The higher Neutral-LogP and Neutral-Mode values in the ASD group indicates that facial expressions in participants with ASD varied less than those in the TD group and appeared more “Neutral” in strength. The higher Happy-LogP and the tendency for a lower Happy-Mode in the ASD group suggests that facial expressions in the ASD participants were more inflexible and tended to appear less “Happy” in strength than those in the TD participants.

**Table 2:** Comparison of EI variables between the autism spectrum disorder (ASD) and typical development (TD) groups.

	ASD (N = 18)		TD (N = 17)				
EI variable	Mean	SD	Mean	SD	*T*-value (*df* = 33)	*P*-value	Cohen's *d*
Neutral-EI							
Mode	0.46	0.06	0.40	0.06	3.03	0.005 ^*1*^	1.02
LogP	2.0	0.4	1.7	0.3	3.21	0.003 ^*1*^	1.08
Happy-EI							
Mode	0.004	0.010	0.047	0.076	−2.26	0.038 ^*2*^	−0.78
LogP	5.7	3.9	2.7	1.7	3.30	0.003 ^*1*^	1.10
Sad-EI							
Mode	0.008	0.012	0.003	0.007	1.46	0.15	0.49
LogP	5.4	3.9	5.9	3.5	−0.34	0.73	−0.12
Angry-EI							
Mode	0.0033	0.0070	0.0026	0.0064	0.31	0.76	0.11
LogP	6.1	4.3	5.9	2.8	0.10	0.92	0.03
Surprised-EI							
Mode	0.017	0.031	0.05	0.11	−0.97	0.35	−0.34
LogP	3.2	1.2	4.0	3.5	−0.90	0.38	−0.31
Scared-EI							
Mode	0.006	0.014	0.018	0.064	−0.72	0.48	−0.25
LogP	5.1	2.2	5.3	2.4	−0.18	0.86	−0.06
Disgusted-EI							
Mode	0.0023	0.0045	0.007	0.013	−1.45	0.16	−0.50
LogP	6.3	4.3	6.2	3.2	0.087	0.93	0.03

^*1*^Significant difference after false discovery rate correction (*P* < 0.05, *P*_FDR_ < 0.05).

^*2*^Difference not significant after false discovery rate correction (*P* < 0.05, *P*_FDR_ > 0.05).

Abbreviations: *EI*, expression intensity; *LogP*, natural logarithm of the probability at the mode of the probability density function; *SD*, standard deviation; *df*, degrees of freedom

Similar analyses using the Means and SDs also indicated that the ASD group had a higher Neutral-Mean, and lower Happy-Mean and Happy-SD values than the TD group (*P*_FDR_ < 0.05, *d* > 1), again indicating that their facial expressions tended to appear more “Neutral” as well as more fixed to a less “Happy” expression than those in the TD group (S4 Table and S2 Fig).

### Correlations between EI variables and ADOS reciprocal social interaction scores

**Fig 2:**
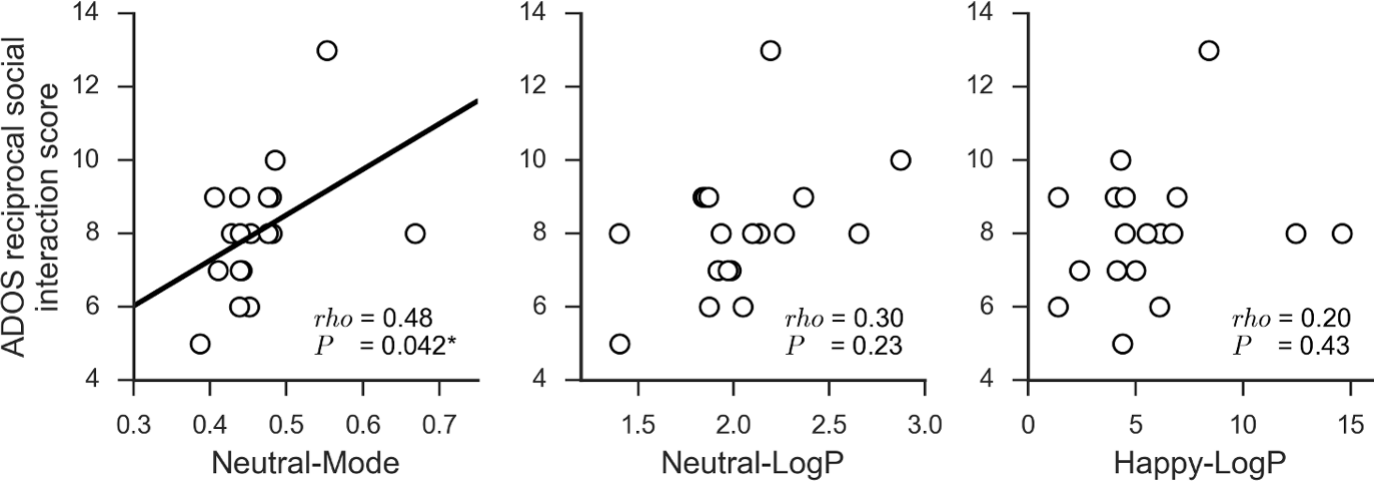
Correlations between EI variables characterizing autism spectrum disorder (ASD) and ADOS reciprocal social interaction scores. Of the three EI variables found to characterize ASD (Fig 1 and Table 2), Neutral-Mode significantly correlated with ADOS reciprocal social interaction scores, whereas Neutral-LogP and Happy-LogP did not. Each circle indicates each participant with ASD. *rho*, Spearman's rank correlation coefficient. *, *P* < 0.05. Abbreviations: *EI*, expression intensity; *ADOS*, Autism Diagnostic Observation Schedule; *LogP*, natural logarithm of the probability at the mode of the probability density function.

We examined the correlations between ADOS reciprocal social interaction scores and the three EI variables that differed significantly between the ASD and TD groups. We found that the scores significantly and positively correlated with Neutral-Mode (*ρ* = 0.48, *P* = 0.042), but not with Neutral-LogP (*P* = 0.23) or Happy-LogP (*P* = 0.39) EI values (Fig 2).

We conducted similar analyses for the three Means/SDs that differed significantly between groups. The results also indicated that social interaction scores significantly and positively correlated with the Neutral-Mean (*ρ* = 0.56, *P* = 0.015], but not with the Happy-Mean (*P* = 0.20) or Happy-SD (*P* = 0.13) EI values (S3 Fig).

### Correlations between EI variables and other clinical/demographic indices

**Fig 3:**
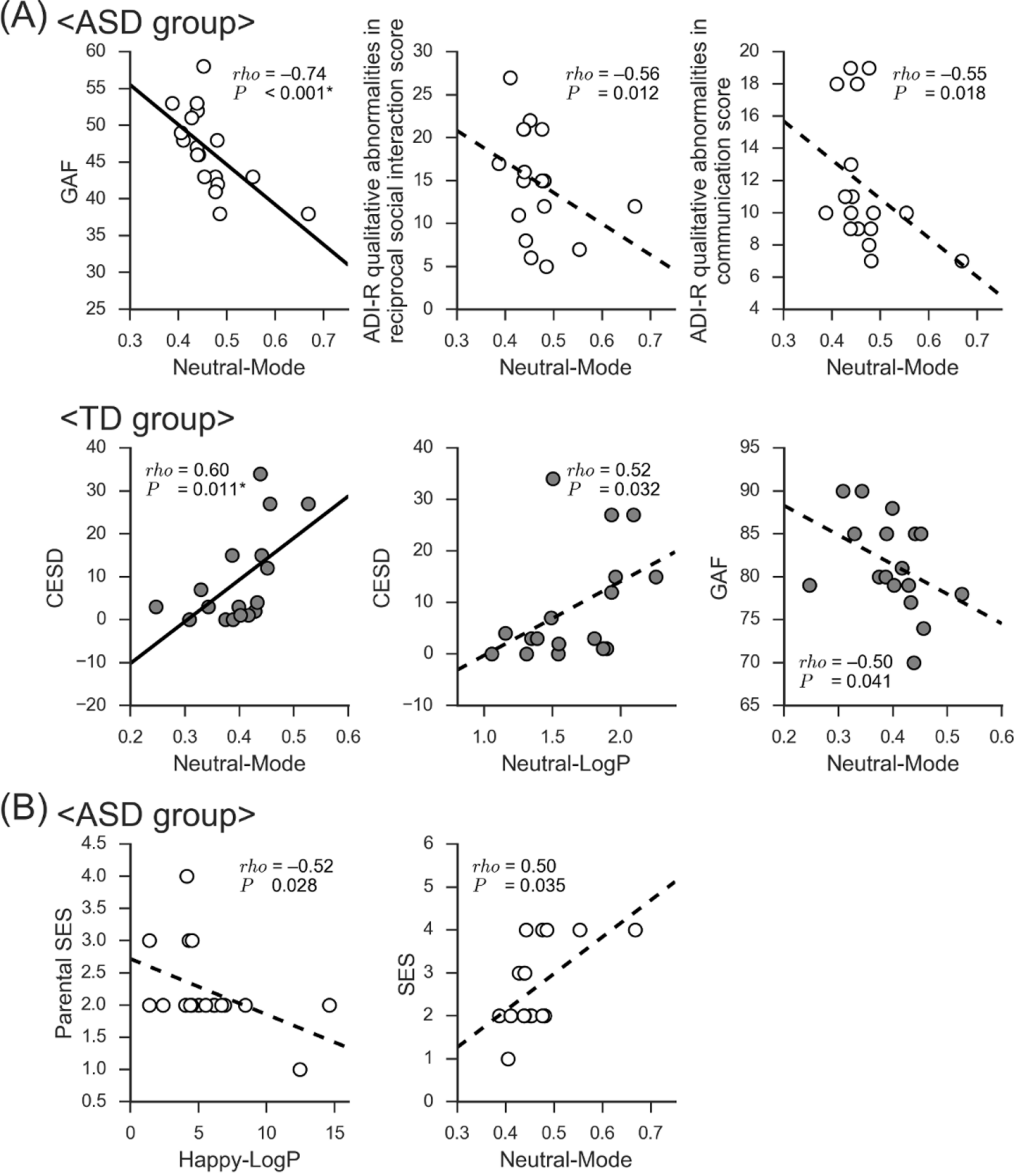
Correlations of EI variables characterizing autism spectrum disorder (ASD) with clinical indices. Correlations of the EI variables found to characterize ASD (Neutral-Mode, Neutral-LogP, and Happy-LogP) with (A) clinical indices (not including ADOS reciprocal social interaction/stereotyped behaviors and restricted interests scores), and (B) demographic indices. Each correlation was evaluated separately for each group. Only the correlations with *P* < 0.05 are shown. (A) Neutral-Mode was significantly correlated with the GAF in the ASD group and with the CESD in the TD group even after FDR correction. (B) No correlation with demographic indices remained significant after FDR correction. Uncolored circles indicate individuals with ASD; colored circles are for those with TD. *rho*, Spearman's rank correlation coefficient. *, FDR-corrected *P* < 0.05. Abbreviations: *EI*, expression intensity; *TD*, typically developing; *GAF*, Global Assessment of Functioning; *ADI-R*, Autism Diagnostic Interview-Revised; *CESD*, Center for Epidemiologic Studies Depression Scale; *SES*, Socioeconomic status; *LogP*, natural logarithm of the probability at the mode of the probability density function; *ADOS*, Autism Diagnostic Observation Schedule.

Correlation analyses between the three identified EI variables (i.e. Neutral-Mode, Neutral-LogP, Happy-LogP) and clinical/demographic indices were performed separately for each group. Among the clinical indices, ADOS stereotyped-behaviors and restricted-interests scores for the ASD group were excluded because the data were binarily distributed (zero or one). Thus, we analyzed correlations between the EI variables and the other clinical indices: STAI-state, CESD, WHOQOL, GAF, and the AQ for both groups; ADOS communication scores, ADI-R qualitative abnormalities in reciprocal social interaction, communication, and restricted, repetitive, and stereotyped patterns of behavior for the ASD group only. The results showed a significant negative correlation between Neutral-Mode EI and GAF scores in the ASD group (*ρ* = −0.74, *P* < 0.001, *P*_FDR_ < 0.05). Similarly, Neutral-Mode EI was also negatively correlated with ADI-R qualitative abnormalities in reciprocal social interaction (*ρ* = −0.51, *P* = 0.036, *P*_FDR_ > 0.05) and with abnormalities in communication (*ρ* = −0.49, *P* = 0.045, *P*_FDR_ > 0.05), although these correlations did not survive correction for multiple comparisons. In the TD group, Neutral-Mode EI was significantly and positively correlated with depressive tendency indexed by the CESD (*ρ* = 0.60, *P* = 0.011, *P*_FDR_ < 0.05). Correlations were also observed between Neutral-LogP and the CESD (*ρ* = 0.52, *P* = 0.032, *P*_FDR_ > 0.05) and between Neutral-Mode and the GAF (*ρ* = −0.50, *P* = 0.041, *P*_FDR_ > 0.05) (Fig 3A), although these correlations were not significant after correcting for multiple comparison. With regard to the demographic indices, Happy-LogP and Neutral-Mode were both correlated with parental SES in the ASD group (Happy-LogP: *ρ* = −0.52, *P* = 0.028, *P*_FDR_ > 0.05; *ρ* = 0.50, *P* = 0.034, *P*_FDR_ > 0.05) (Fig 3B).

When similar analyses were performed using the Means/SDs, none of the correlations between the three EI variables and the clinical/demographic indices remained significant after FDR correction, although the following correlations were detected at *P* < 0.05: Neutral-Mean and the GAF in the ASD group (*ρ* = −0.55, *P* = 0.017, *P*_FDR_ > 0.05); Neutral-Mean and the AQ in the TD group (*ρ* = 0.52, *P* = 0.033, *P*_FDR_ > 0.05); Happy-Mean and parental SES (*ρ* = 0.56, *P* = 0.016, *P*_FDR_ > 0.05); Happy-SD and parental SES (*ρ* = 0.50, *P* = 0.035, *P*_FDR_ > 0.05) in the ASD group (S4 Fig).

## Discussion

The current study showed the following two main findings. First, facial expressions in the participants with ASD appeared more “Neutral” and less “Happy” than those in TD controls, indicating that facial expressivity in those with ASD was attenuated in socially interactive situations. Second, the greater “Neutral” intensity in people with ASD correlated well with their impairment in social reciprocity, as indexed by the ADOS reciprocal social interaction score.

Despite the fact that clinical knowledge for low facial expressivity in ASD has been well-established [25], to our knowledge, this is the first study to detect this autistic feature under social interactive situations without any specific task intended to explicitly elicit particular emotional facial expressions. While several case-control studies have reported incongruous mimicry [18, 20, 21] with large effect sizes (*d* = 0.8−1.34) [13], and concluded that people with ASD exhibit abnormalities in some elements of facial expression [18, 20, 21], other studies that subjectively evaluated Neutral facial expression could not find any significant differences between ASD and TD groups of individuals [16, 24, 25]. Therefore, the ability to detect poor facial expressivity with a relatively large effect size in the current study (*d* = 1.02−1.10) might be related to the advantages that computerized FEA is superior to other methods. In particular, the computerized FEA is able to individually evaluate each facial expression element, rather than needing to categorize the facial expressions into a representative expression. This allowed us to objectively measure facial expression intensity values at precise resolution.

The positive correlation between higher Neutral-Mode intensity value and higher ADOS reciprocal social interaction score suggests that a reduction of facial expressivity predicts the severity of a core social symptom of ASD, and is partially consistent with a study that reported a significant correlation between reduced facial mimicry measured by FACS and social dysfunction evaluated by the Childhood Autism Rating Scale [21]. Although the ADOS reciprocal social interaction domain consists of several items designed to evaluate non-verbal communications including proper usage of facial expressions, the rater needs to administer all 15 of the ADOS activities to score this domain. In contrast, the current computerized quantitative FEA is applicable for short subsets of the ADOS, even when they are less than one minute in duration. Therefore, our method might be able to evaluate impairments in social reciprocity in a much shorter time than the standard full ADOS, and will thus make repeatable measurements easier to obtain.

Our results also indicated significant correlations between Neutral expression intensity and several other clinical/demographic indices such as the GAF in people with ASD and the CESD in people with TD. These correlations indicate the possibility that an increase in Neutral facial expression could reflect a worsening clinical/psychofunctional status in addition to impairment in social reciprocity. For people with ASD, individual differences in facial expressivity might relate to their general ability to function in daily life, while for those with TD, it might just relate to mood and emotional status.

Happy-LogP intensity was also higher in the ASD group than in the TD group, although it was not correlated with any other clinical/demographic index. To understand these results, the distribution of the Happy-Mode values should be taken into consideration (Fig 1). In contrast to the fairly normal distribution of Neutral-Mode EI values, the Happy-Mode distribution for each participant was mostly near the bottom (especially in the ASD group), reflecting sharply and positively skewed probability density curves for the Happy EI values. Thus, Happy facial expressions almost never appeared in most participants with ASD, and occasionally appeared in a few. Additionally, as both Mode and LogP are derived from the same probability density curve of EI values, the participants with higher Happy-LogP tended to exhibit fewer “Happy” facial expressions under this circumstance. Therefore, although the current study successfully showed low Happy facial expression in the ASD group, which is in line with several previous studies [20, 21], further studies into the relationship between Happy facial expression and clinical/demographic indices beyond group differences should include experimental designs that are optimized so as to decrease the extreme skewness in the Happy EI-value distribution.

The present study has several potential limitations and methodological considerations. First, the sample size might have limited the detectability of autistic features in the other facial expression elements (i.e., Surprised, Sad, Scared, Angry, and Disgusted). Second, while the racial uniformity of the participants was desirable for the software to apply a proper facial model and maintain analytical consistency, cultural uniformity also should be taken into consideration when applying these research findings to other populations because facial expressions are known to differ across cultures [41, 42].

In conclusion, lower than normal facial expressivity indicated by an abnormally high Neutral facial expression during social interactions could be an objective, repeatable, and sensitive marker for core social symptoms of ASD. Further, measuring this autistic feature via computerized quantitative FEA is likely to be a promising and reliable method.

## Supporting information

S1 Fig Representative facial expression intensity (EI) charts output by FaceReader. Representative time-series expression intensity charts for each of the seven expressions that were output by FaceReader software are shown for a participant with typical development (A) and a participant with autism spectrum disorder (B). Note that the sum of the intensity values at a given point is normally not equal to one because FaceReader usually evaluates a facial expression as a mixture of several neutral/emotional expressions.
(TIFF)

S2 Fig Comparison of EI variables (Mean/SD of Neutral/Happy EI values) between the autism spectrum disorder (ASD) and typical development (TD) groups. When the Means and SDs of EI values were employed as EI variables, the ASD group showed significantly higher Neutral-Mean, and significantly lower Happy-Mean and Happy-SD than the TD group, with effect sizes (*P*_FDR_ < 0.05, *d* > 1 or *d* < −1) that were just as large as those for Neutral-Mode, Neutral-LogP, and Happy-LogP (Fig 1 and Table 2). Happy-Mode did not differ significantly between the groups. Each dot represents each participant. The horizontal and vertical bars show mean values and 95% confidence intervals respectively. *d*, Cohen’s *d*. *, *P*_FDR_ < 0.05. Abbreviations: *EI*, expression intensity; *SD*, standard deviation; *LogP*, natural logarithm of the probability at the mode of the probability density function.
(TIFF)

S3 Fig Correlations of EI variables (Mean/SD) characterizing autism spectrum disorder (ASD) with ADOS reciprocal social interaction scores. Of the three EI variables (Mean/SD) that differed significantly between groups (S2 Fig and S4 Table), Neutral-Mean was significantly correlated with ADOS reciprocal social interaction scores, but Happy-Mean and Happy-SD were not. *rho*, Spearman's rank correlation coefficient. *, *P* < 0.05. Abbreviations: *EI*, expression intensity; *ADOS*, Autism Diagnostic Observation Schedule; *SD*, standard deviation.
(TIFF)

S4 Fig Correlations of EI variables (Mean/SD) characterizing autism spectrum disorder (ASD) with clinical/demographic indices. Correlations of the EI variables (Mean/SD) that characterized ASD (Neutral-Mean, Happy-Mean, and Happy-SD) with clinical indices (A) (not including the Autism Diagnostic Observation Schedule reciprocal social interaction/stereotyped behaviors and restricted interests scores), and demographic indices (B), evaluated separately for each group. Although several correlations were significant at *P* < 0.05, none remained significant after FDR correction. Uncolored circles indicate individuals with ASD; colored circles are for those with TD. rho, Spearman's rank correlation coefficient. *, FDR-corrected *P* < 0.05. Abbreviations: *EI*, expression intensity; *GAF*, Global Assessment of Functioning; *AQ*, Autism Spectrum Quotient; *SES*, Socioeconomic status; *SD*, standard deviation.
(TIF)

S1 Table Assessments of structure and repeatability for each Autism Diagnostic Observation Schedule (ADOS) activity. (DOCX)

S2 Table Quantitative evaluation (Z-score) of the feasibility of facial expression analysis for the ADOS activities. (DOCX)

S3 Table Correlations between EI variables (Mode vs. Mean and LogP vs. SD) for each facial expression element. (DOCX)

S4 Table Comparison of EI variables (Mean/SD) between the autism spectrum disorder (ASD) and typically developing (TD) groups. (DOCX)

S1 Data set The minimal data set necessary to replicate our study findings. (CSV)
