# Supplementary figures and images for: Computer-analyzed facial expression as a surrogate marker for autism spectrum social core symptoms
Source: PLoS One. 2018 Jan 2;13(1):e0190442. doi: 10.1371/journal.pone.0190442 (PMC5749804; doi:10.1371/journal.pone.0190442)

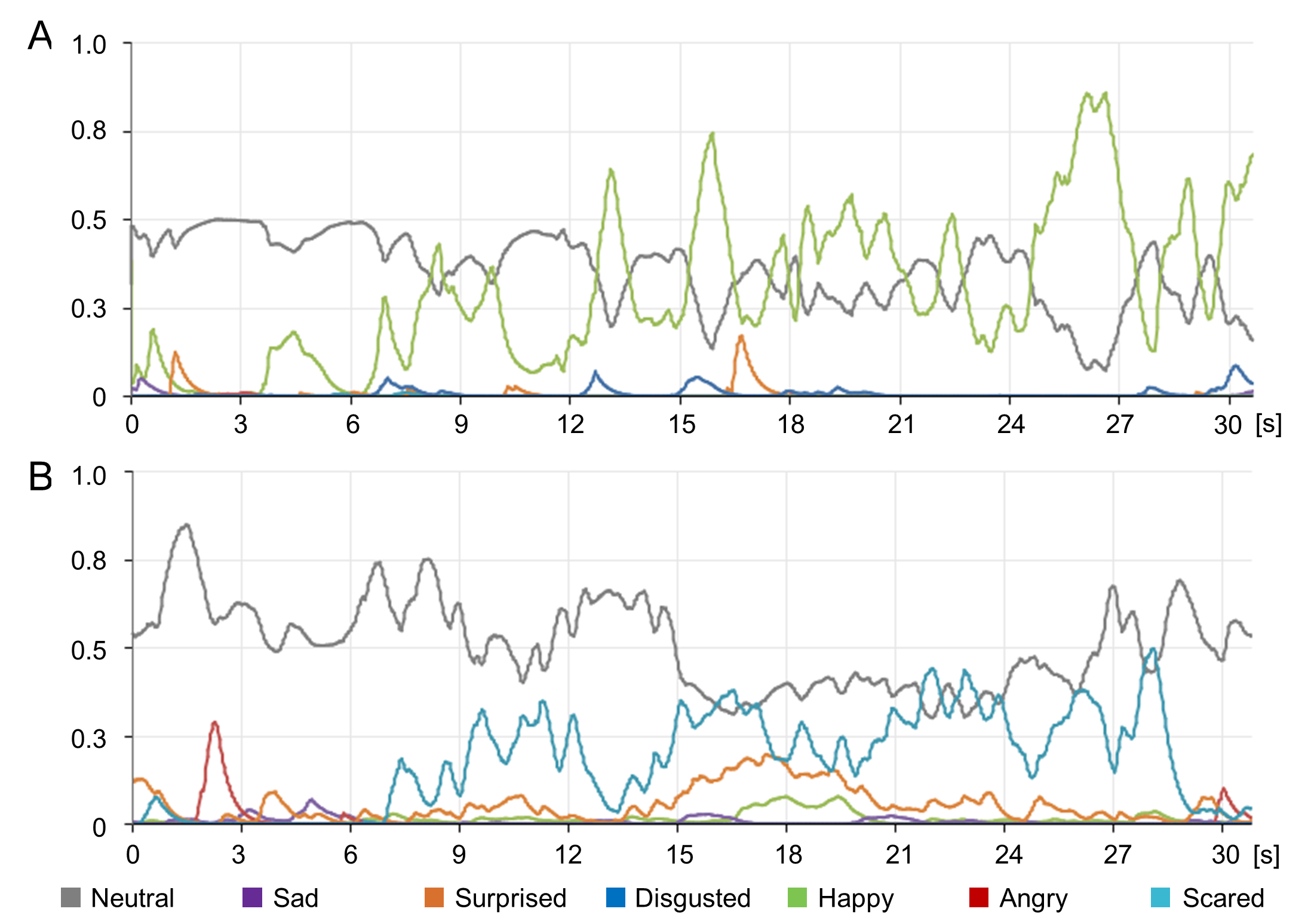

Supplement: S1 Fig — Representative time-series expression intensity charts for each of the seven expressions that were output by FaceReader software are shown for a participant with typical development (A) and a participant with autism spectrum disorder (B). Note that the sum of the intensity values at a given point is normally not equal to one because FaceReader usually evaluates a facial expression as a mixture of several neutral/emotional expressions. (TIFF) [file pone.0190442.s001.tiff]

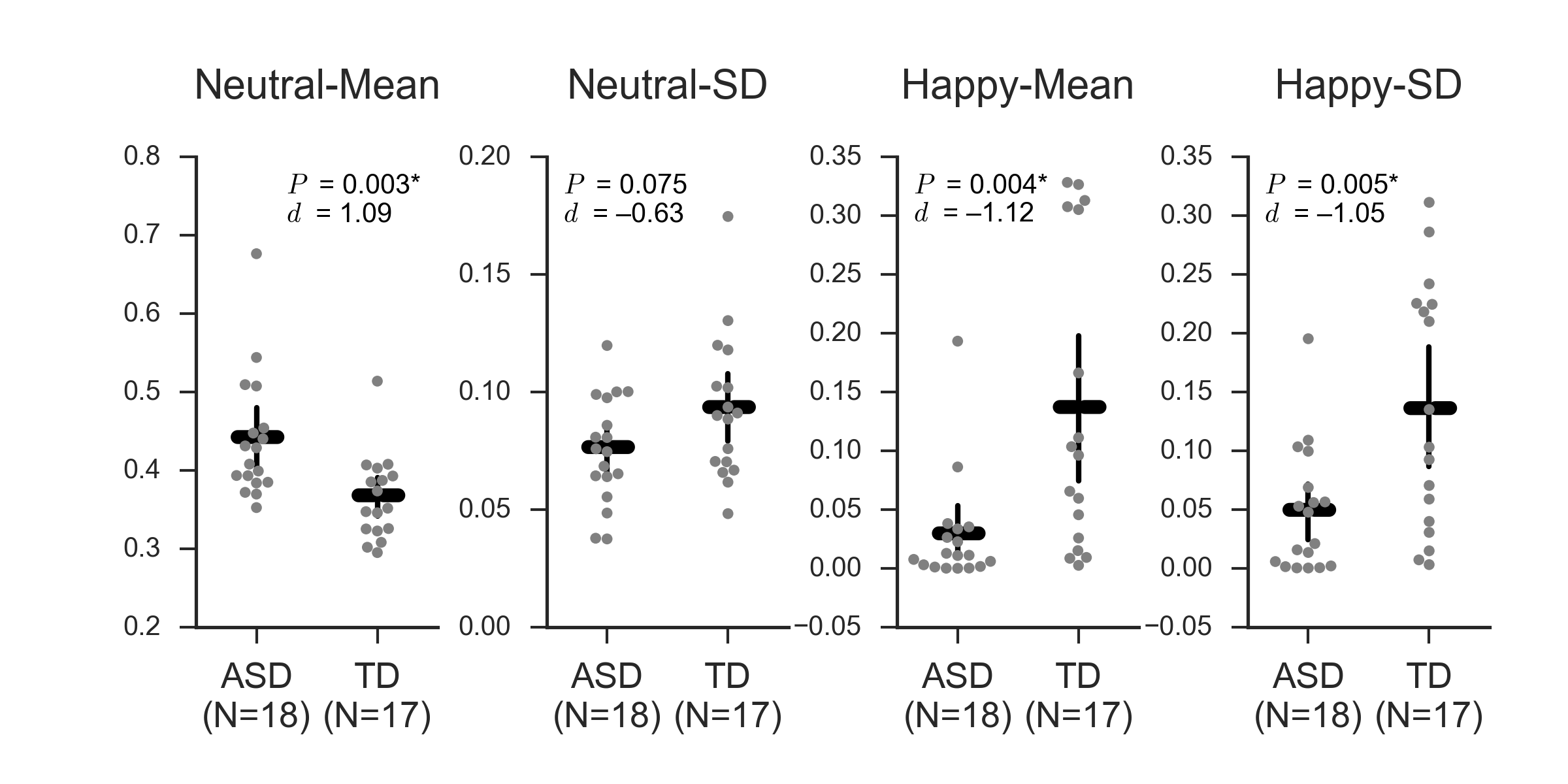

Supplement: S2 Fig — When the Means and SDs of EI values were employed as EI variables, the ASD group showed significantly higher Neutral-Mean, and significantly lower Happy-Mean and Happy-SD than the TD group, with effect sizes (PFDR < 0.05, d > 1 or d < −1) that were just as large as those for Neutral-Mode, Neutral-LogP, and Happy-LogP (Fig 1 and Table 2). Happy-Mode did not differ significantly between the groups. Each dot represents each participant. The horizontal and vertical bars show mean values and 95% confidence intervals respectively. d, Cohen’s d. *, PFDR < 0.05. Abbreviations: EI, expression intensity; SD, standard deviation; LogP, natural logarithm of the probability at the mode of the probability density function. (TIFF) [file pone.0190442.s002.tiff]

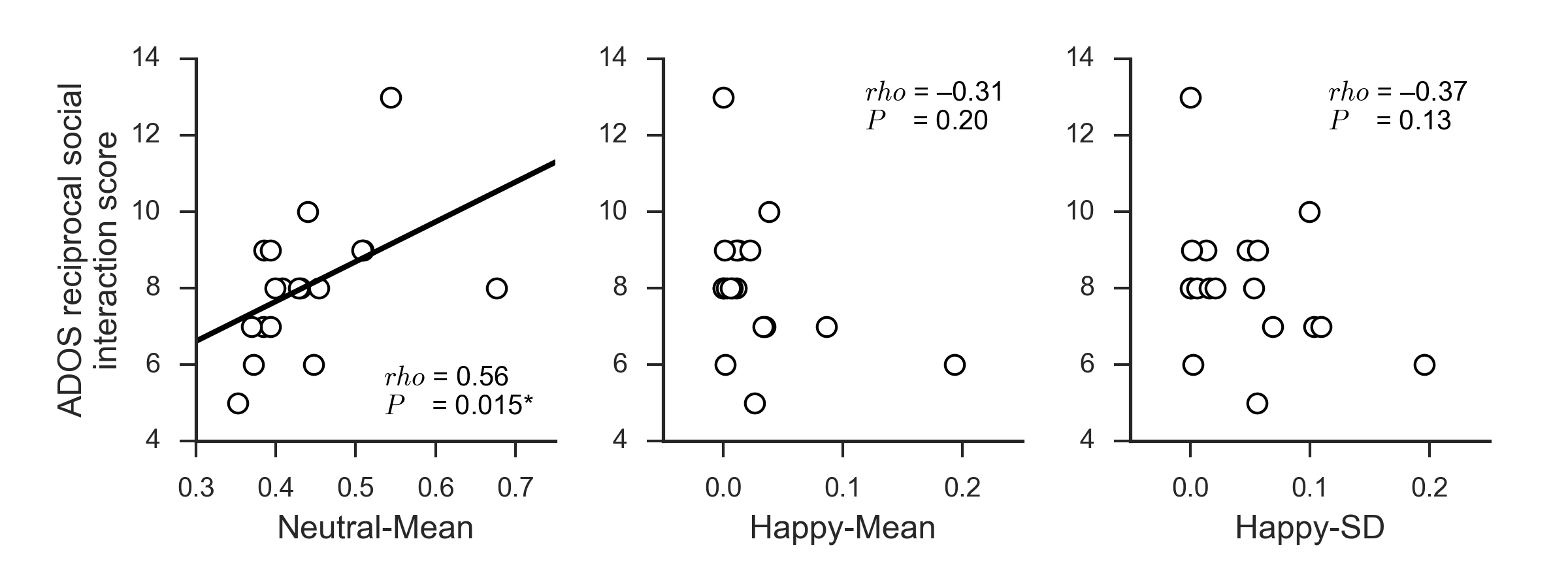

Supplement: S3 Fig — Of the three EI variables (Mean/SD) that differed significantly between groups (S2 Fig and S4 Table), Neutral-Mean was significantly correlated with ADOS reciprocal social interaction scores, but Happy-Mean and Happy-SD were not. rho, Spearman's rank correlation coefficient. *, P < 0.05. Abbreviations: EI, expression intensity; ADOS, Autism Diagnostic Observation Schedule; SD, standard deviation. (TIFF) [file pone.0190442.s003.tiff]

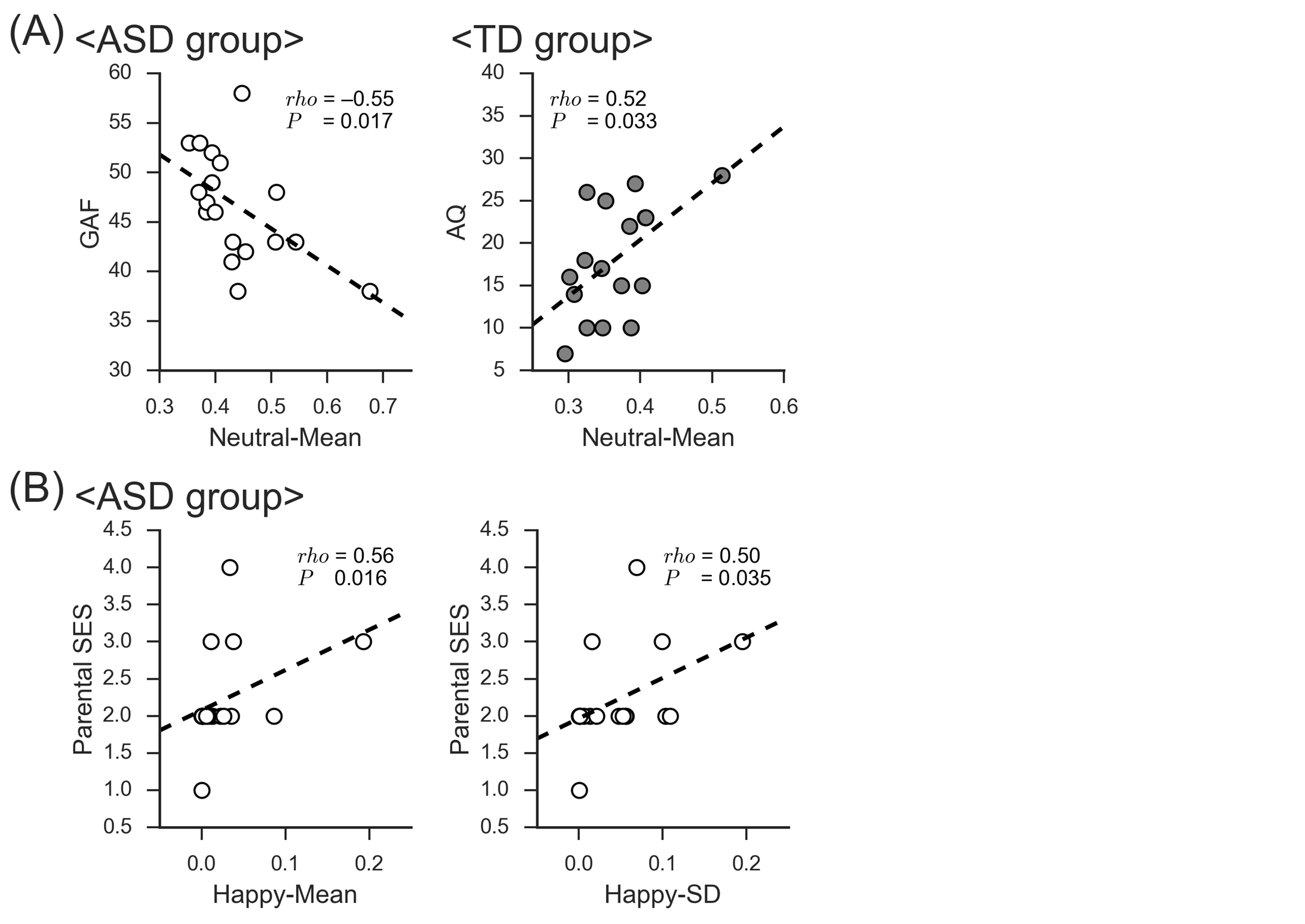

Supplement: S4 Fig — Correlations of the EI variables (Mean/SD) that characterized ASD (Neutral-Mean, Happy-Mean, and Happy-SD) with clinical indices (A) (not including the Autism Diagnostic Observation Schedule reciprocal social interaction/stereotyped behaviors and restricted interests scores), and demographic indices (B), evaluated separately for each group. Although several correlations were significant at P < 0.05, none remained significant after FDR correction. Uncolored circles indicate individuals with ASD; colored circles are for those with TD. rho, Spearman's rank correlation coefficient. *, FDR-corrected P < 0.05. Abbreviations: EI, expression intensity; GAF, Global Assessment of Functioning; AQ, Autism Spectrum Quotient; SES, Socioeconomic status; SD, standard deviation. (TIF) [file pone.0190442.s004.tif]
