# Supplementary material for: Computer-analyzed facial expression as a surrogate marker for autism spectrum social core symptoms
Source: PLoS One. 2018 Jan 2;13(1):e0190442. doi: 10.1371/journal.pone.0190442 (PMC5749804; doi:10.1371/journal.pone.0190442)
Supplement: S1 Table — (DOCX) [file pone.0190442.s005.docx]

**S1 Table. Assessments of structure and repeatability for each Autism Diagnostic Observation Schedule (ADOS) activity**

| Activity | Context | Structuration | Repeatability |
| --- | --- | --- | --- |
| Construction task | The participant assembles blocks to construct a design shown on a printed form. | ○ | ○ |
| Telling a story from a book | The participant recounts a sequential story from a book of pictures and comments about social relationships and affect. | ○ | ○ |
| Description of a picture | The participant looks at a picture and comments on the picture spontaneously. | × | ○ |
| Conversation | The participant has a conversation and recounts routine or nonroutine events (e.g., a birthday party, a vacation). | × | ○ |
| Current work/school | The participant describes his current situation, his insight about everyday situations, and his understanding about role in determining what will happen in the future. | ○ | × |
| Social difficulties/annoyance | The participant talks about his insight into personal social difficulties and sense of responsibility for his own actions in response to administrator's questions. | ○ | × |
| Emotions | Participant talks about emotions and personal experiences in response to administrator's questions | ○ | × |
| Demonstration task | The participant demonstrates toothbrushing using gesture or mime with accompanying language. | ○ | ○ |
| Cartoons | Participant narrates a story, uses gesture to enact events, and integrates gesture with gaze and language and comment on the emotions of the characters in the stories. | ○ | ○ |
| Break | The participant has a break and may be interested in materials provided for the break. | × | ○ |
| Daily living | The participant talks about factual information and background regarding money, residential arrangements, and leisure activities in response to administrator's questions. | ○ | × |
| Friends/marriage | The participant talks about one or more relationships that the participant would consider to be friendships, and describes his understanding of the concept of friendships and the idea of getting married in response to administrator's questions. | ○ | × |
| Loneliness | The participant talks about his social situation and emotional reaction to it in response to administrator's questions. | ○ | × |
| Plans and dreams | The participant talks about any goals that he may have. | ○ | × |
| Creating a story | The participant chooses five objects and makes up a story, newscast, or commercial. | × | ○ |
