# Supplementary material for: Computer-analyzed facial expression as a surrogate marker for autism spectrum social core symptoms
Source: PLoS One. 2018 Jan 2;13(1):e0190442. doi: 10.1371/journal.pone.0190442 (PMC5749804; doi:10.1371/journal.pone.0190442)
Supplement: S2 Table — (DOCX) [file pone.0190442.s006.docx]

**S2 Table. Quantitative evaluation (Z-score) of the feasibility of facial expression analysis for the ADOS activities**

| ADOS activity and types of Z-score *^1^* | N | Mean | SD | *T-*value | *P-*value |
| --- | --- | --- | --- | --- | --- |
| *Cartoons* |  |  |  |  |  |
| Total | 18 | 2.25 | 1.99 |  |  |
| Proportion of success in face recognition | 18 | 0.27 | 1.12 |  |  |
| Activity length | 18 | 0.50 | 0.08 |  |  |
| Validity of head orientations | 18 | 0.83 | 0.57 |  |  |
| Reliability of head orientations | 18 | 0.65 | 0.78 |  |  |
| *Construction task* |  |  |  |  |  |
| Total | 17 | −0.69 | 1.13 | 5.25  (*df* = 33) ^2^ | < 0.001 *^3^* |
| Proportion of success in face recognition | 17 | −0.16 | 0.83 |  |  |
| Activity length | 17 | 0.33 | 0.10 |  |  |
| Validity of head orientations | 17 | −0.44 | 0.74 |  |  |
| Reliability of head orientations | 17 | −0.42 | 0.63 |  |  |
| *Telling a story from a book* |  |  |  |  |  |
| Total | 16 | −1.79 | 2.89 | 4.55  (*df* = 32) ^2^ | < 0.001 *^3^* |
| Proportion of success in face recognition | 16 | −0.14 | 0.92 |  |  |
| Activity length | 16 | −0.91 | 1.37 |  |  |
| Validity of head orientations | 16 | −0.46 | 1.00 |  |  |
| Reliability of head orientations | 16 | −0.29 | 1.12 |  |  |

*^1^*Z-scores are plus-signed in favorable directions (i.e., shorter activity length, higher proportion of successful face recognition, higher validity, and higher reliability).

*^2^*Compared by one-tailed *T*-test with the sum of Z-scores for the *Cartoons* activity.

*^3^*Compared with the summed Z-scores for the *Cartoons* activity, significant after false discovery rate correction (*P* < 0.05, *P*_FDR_ < 0.05).

Abbreviations: *ADOS*, Autism Diagnostic Observation Schedule; *LogP*, natural logarithm of the probability at the mode of the probability density function; *SD*, standard deviation; *df*, degrees of freedom.
