# Supplementary material for: Computer-analyzed facial expression as a surrogate marker for autism spectrum social core symptoms
Source: PLoS One. 2018 Jan 2;13(1):e0190442. doi: 10.1371/journal.pone.0190442 (PMC5749804; doi:10.1371/journal.pone.0190442)
Supplement: S3 Table — (DOCX) [file pone.0190442.s007.docx]

**S3 Table. Correlations between EI variables (Mode vs. Mean and LogP vs. SD) for each facial expression element**

|  | Mode vs. Mean | | LogP vs. SD | |
| --- | --- | --- | --- | --- |
| Element of facial expression (N = 35) | Spearman's *ρ* | *P-*value | Spearman's *ρ* | *P-*value |
| Neutral | 0.87 | < 0.001 ^1^ | −0.84 | < 0.001 ^1^ |
| Happy | 0.91 | < 0.001 ^1^ | −0.86 | < 0.001 ^1^ |
| Sad | 0.83 | < 0.001 ^1^ | −0.77 | < 0.001 ^1^ |
| Angry | 0.53 | 0.001 ^1^ | −0.40 | 0.018 ^1^ |
| Surprised | 0.72 | < 0.001 ^1^ | −0.58 | < 0.001 ^1^ |
| Scared | 0.78 | < 0.001 ^1^ | −0.64 | < 0.001 ^1^ |
| Disgusted | 0.64 | < 0.001 ^1^ | −0.36 | 0.034 ^1^ |
