# Supplementary material for: Computer-analyzed facial expression as a surrogate marker for autism spectrum social core symptoms
Source: PLoS One. 2018 Jan 2;13(1):e0190442. doi: 10.1371/journal.pone.0190442 (PMC5749804; doi:10.1371/journal.pone.0190442)
Supplement: S4 Table — (DOCX) [file pone.0190442.s008.docx]

**S4 Table. Comparison of EI variables (Mean/SD) between the autism spectrum disorder (ASD) and typically developing (TD) groups.**

|  | ASD (N = 18) | | TD (N = 17) | |  |  |  |
| --- | --- | --- | --- | --- | --- | --- | --- |
| EI variable | Mean | SD | Mean | SD | *T-*value (*df* = 33) | *P-*value | Cohen's *d* |
| Neutral-EI |  |  |  |  |  |  |  |
| Mean | 0.44 | 0.08 | 0.36 | 0.052 | 3.25 | 0.003 *^1^* | 1.09 |
| SD | 0.075 | 0.022 | 0.092 | 0.030 | −1.84 | 0.075 | −0.63 |
| Happy-EI |  |  |  |  |  |  |  |
| Mean | 0.027 | 0.045 | 0.13 | 0.12 | −3.26 | 0.004 *^1^* | −1.13 |
| SD | 0.047 | 0.051 | 0.13 | 0.10 | −3.06 | 0.005 *^1^* | −1.05 |
| Sad-EI |  |  |  |  |  |  |  |
| Mean | 0.049 | 0.047 | 0.033 | 0.040 | 1.10 | 0.28 | 0.37 |
| SD | 0.069 | 0.049 | 0.054 | 0.040 | 0.96 | 0.35 | 0.32 |
| Angry-EI |  |  |  |  |  |  |  |
| Mean | 0.029 | 0.029 | 0.026 | 0.034 | 0.30 | 0.77 | 0.10 |
| SD | 0.044 | 0.032 | 0.047 | 0.046 | −0.23 | 0.82 | −0.08 |
| Surprised-EI |  |  |  |  |  |  |  |
| Mean | 0.064 | 0.060 | 0.081 | 0.070 | −0.77 | 0.45 | −0.26 |
| SD | 0.072 | 0.053 | 0.087 | 0.048 | −0.84 | 0.40 | −0.28 |
| Scared-EI |  |  |  |  |  |  |  |
| Mean | 0.027 | 0.036 | 0.039 | 0.088 | −0.43 | 0.67 | −0.15 |
| SD | 0.036 | 0.036 | 0.038 | 0.056 | −0.15 | 0.88 | −0.05 |
| Disgusted-EI |  |  |  |  |  |  |  |
| Mean | 0.023 | 0.031 | 0.031 | 0.042 | −0.62 | 0.54 | −0.21 |
| SD | 0.043 | 0.048 | 0.041 | 0.050 | 0.11 | 0.92 | 0.04 |
